# JAM-A regulates cortical dynein localization through Cdc42 to control planar spindle orientation during mitosis

**DOI:** 10.1038/ncomms9128

**Published:** 2015-08-26

**Authors:** Hüseyin Tuncay, Benjamin F. Brinkmann, Tim Steinbacher, Annika Schürmann, Volker Gerke, Sandra Iden, Klaus Ebnet

**Affiliations:** 1 Institute-Associated Research Group ‘Cell Adhesion and Cell Polarity', University of Münster, 48149 Münster, Germany; 2 Institute of Medical Biochemistry, ZMBE, University of Münster, 48149 Münster, Germany; 3 Interdisciplinary Clinical Research Center (IZKF), University of Münster, 48149 Münster, Germany; 4 Cells-in-Motion Cluster of Excellence (EXC 1003—CiM), University of Münster, 48149 Münster, Germany; 5 Present address: Cologne Excellence Cluster CECAD, University Hospital Cologne, 50931 Cologne, Germany

## Abstract

Planar spindle orientation in polarized epithelial cells depends on the precise localization of the dynein–dynactin motor protein complex at the lateral cortex. The contribution of cell adhesion molecules to the cortical localization of the dynein–dynactin complex is poorly understood. Here we find that junctional adhesion molecule-A (JAM-A) regulates the planar orientation of the mitotic spindle during epithelial morphogenesis. During mitosis, JAM-A triggers a transient activation of Cdc42 and PI(3)K, generates a gradient of PtdIns(3,4,5)P3 at the cortex and regulates the formation of the cortical actin cytoskeleton. In the absence of functional JAM-A, dynactin localization at the cortex is reduced, the mitotic spindle apparatus is misaligned and epithelial morphogenesis in three-dimensional culture is compromised. Our findings indicate that a PI(3)K- and cortical F-actin-dependent pathway of planar spindle orientation operates in polarized epithelial cells to regulate epithelial morphogenesis, and we identify JAM-A as a junctional regulator of this pathway.

## Abstract (web summary)

Polarized epithelial cells orient their mitotic spindles in the plane of the sheet but the role of cell adhesion molecules in this process is poorly understood. Here Tuncay *et al.* show that JAM-A regulates spindle orientation by creating a gradient of PtdIns(3,4,5)P_3_, regulating cortical actin assembly and localizing dynactin to the cell cortex.

## Graphical abstract

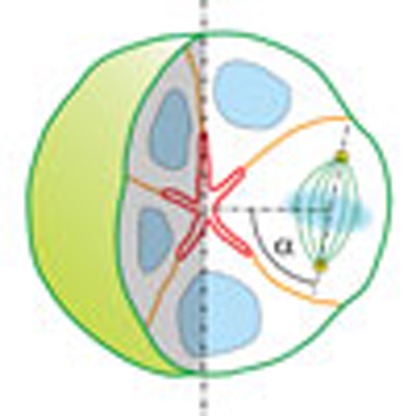

The orientation of cell division is tightly regulated to ensure proper tissue morphogenesis and to prevent cancerous growth. Cell division can be symmetric resulting in two equal daughter cells and also asymmetric resulting in two daughter cells with different fates^1^. In both cases, the orientation of the cell division axis is regulated by dynamic anchoring of the mitotic spindle at the cell cortex through astral microtubules (MT) that emanate from the centrosomes. Astral MTs have been proposed to mediate spindle positioning by generating pulling forces by way of the MT minus end-directed dynein–dynactin motor protein complex (hereafter referred to as dynein for simplicity)^2^. Dynein at the cortex can capture cortex-sampling astral MTs, and through its motor protein activity it can generate tension on the centrosomes resulting in torque on the mitotic apparatus until the astral MTs reach cortical sites with maximum levels of dynein-binding proteins^3^.

In epithelial cells of higher eukaryotes, dynein interacts with the protein Nuclear Mitotic Apparatus (NuMA)^4^, which forms a ternary complex with Leu-Gly-Asn repeat-enriched protein (LGN) and Gαi (NuMA–LGN–Gαi complex and Mud–Pins–Gαi complex in *Drosophila*). In this complex LGN acts as a linker between NuMA and myristoylated, membrane-anchored Gαi-GDP^5^ and all three components are required to regulate planar spindle orientation during epithelial morphogenesis *in vitro*^6^. The polarity proteins Par3 and aPKC direct the localization of LGN to the lateral cell cortex during symmetric cell division, thus ensuring planar spindle orientation in polarized epithelial cells^7^. Studies in non-polarizing HeLa cells indicate that the stable localization of dynein at the lateral cortex requires the activity of Cdc42, which acts through a pathway that involves phosphatidylinositol-3,4,5-triphosphate (PtdIns(3,4,5)P3) and the actin cytoskeleton^8^,^9^,^10^. This pathway was identified in single HeLa cells in the absence of cell–cell contacts and was found not to operate in polarized Madin-Darby Canine Kidney (MDCK) epithelial cells^9^.

Signals originating at the cell–cell contact sites and initiated by cell adhesion molecules are likely to provide spatial cues for the correct localization of dynein at the cortex and to contribute to planar spindle orientation^11^. Evidence exists, suggesting that these cortical cues can regulate spindle orientation independently of LGN and NuMA, as downregulation of LGN or NuMA in asymmetrically dividing basal progenitor cells in the skin switches spindle alignment from apico-basal to planar orientation rather than randomizing spindle orientation^12^. The nature of the signal-providing adhesion molecules and the molecular pathways initiated by them are unclear.

Junctional adhesion molecule (JAM)-A is an immunoglobulin superfamily member localized at intercellular contacts of epithelial and endothelial cells^13^. During cell–cell contact formation, JAM-A recruits the PAR–aPKC complex to primordial adherens junctions where JAM-A is phosphorylated by aPKC at Ser285, to promote the maturation of intercellular junctions and the development of functional tight junctions (TJs)^14^,^15^,^16^. In fully polarized epithelial cells JAM-A Ser285 phosphorylation is restricted to TJs and a non-phosphorylated pool of JAM-A molecules resides beneath the TJs along the lateral cell–cell contacts^16^. The unknown function of this lateral JAM-A pool prompted us to address the question whether JAM-A plays a role during mitosis. Here we describe that JAM-A knockdown or expression of a dimerization-deficient, dominant-negative mutant of JAM-A results in aberrant spindle orientation. JAM-A activates Cdc42 during mitosis, stimulates phosphoinositide 3-kinase (PI(3)K) activation and PtdIns(3,4,5)P3 accumulation at the midcortex, regulates the formation of the cortical actin cytoskeleton and is required for the cortical localization of dynein during mitosis. Our findings establish the Cdc42–PI(3)K–cortical actin-dependent planar spindle orientation pathway in polarized epithelial cells and identify JAM-A as junctional regulator of this pathway.

## Results

### JAM-A regulates planar spindle orientation in MDCK cells

**Figure 1:**
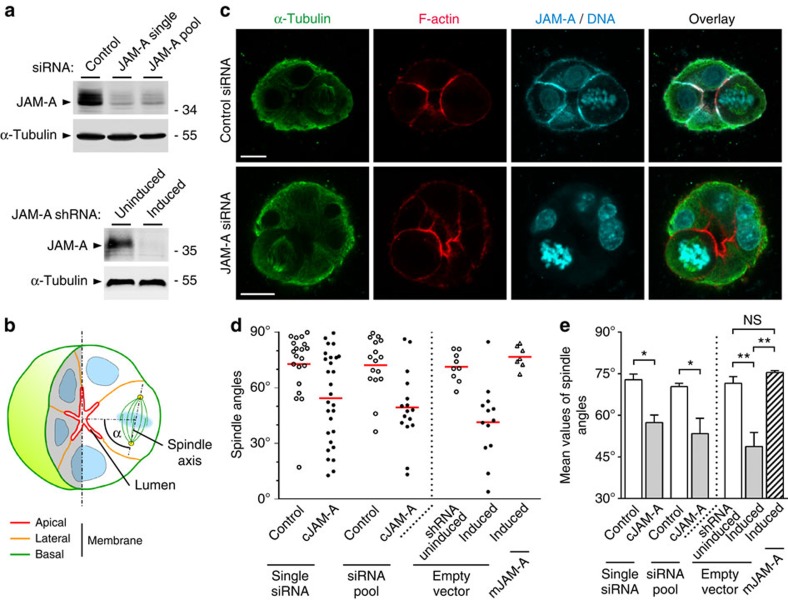
JAM-A regulates spindle orientation in MDCK cells. (**a**) Efficiencies of JAM-A knockdown in MDCK cells either transiently transfected with siRNAs (single siRNA or siRNA pool, top panel) or stably transfected with a doxycycline-regulated JAM-A shRNA expression vector (bottom panel). Knockdown efficiencies typical for a single experiment are shown. (**b**) Schematic illustration of spindle orientation analysis in 3D-cultured MDCK cells. (**c**) MDCK cells transfected with control siRNAs or JAM-A siRNAs were grown in 3D collagen gels and stained for α-tubulin (green), F-actin (apical membrane, red) and DNA/JAM-A (blue) as indicated. It is noteworthy that the mitotic spindle (α-tubulin) aligns parallel to the apical membrane in control cells but almost perpendicular to the apical membrane in JAM-A knockdown cells. Size bars, 10 μm. (**d**) Representative scatter diagrams of single experiments for JAM-A knockdown cells using either single siRNAs or siRNA pools (left of dotted line) directed against canine JAM-A (cJAM-A) or using inducible cJAM-A shRNAs in the absence or the presence of murine JAM-A (mJAM-A) cDNA expression vectors (right of dotted line). (**e**) Statistical analysis of spindle orientation in JAM-A knockdown cells (left of dotted line) and JAM-A knockdown cells expressing mouse JAM-A (right of dotted line). Quantification of data was performed from three independent experiments in each condition and is presented as means±s.e.m.; ns, not significant; *P<0.05, **P<0.01. Data sets containing two groups were analysed using unpaired Student's *t*-test, data sets containing more than two groups were analysed using one-way ANOVA with Tukey's *post-hoc* test.

**Figure 2:**
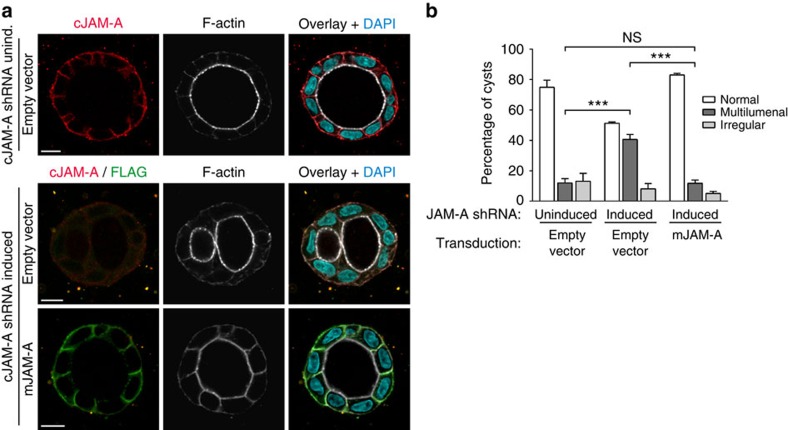
JAM-A regulates single lumen specification in MDCK cells. (**a**) MDCK cells expressing JAM-A shRNAs under a doxycycline-regulated promoter were transduced with lentiviral vectors expressing murine Flag-tagged JAM-A (mJAM-A). Cells were grown in 3D collagen gels for 6–8 days and stained as indicated. Size bars, 10 μm. It is noteworthy that JAM-A knockdown results in a multilumenal phenotype, and that single lumen formation is restored on expression of murine JAM-A. (**b**) Statistical analysis of lumen formation in JAM-A knockdown cells transduced with either empty vector or murine Flag-JAM-A. Quantification of data shown in this figure was performed using one-way ANOVA with Tukey's *post-hoc* test, with three independent experiments in each condition, and is presented as means±s.e.m.; ns, not significant, ***P<0.001.

To address the question whether JAM-A regulates the orientation of the mitotic spindle in polarized epithelial cells, we inhibited JAM-A expression with short interfering RNAs (siRNAs) and analysed spindle orientation during epithelial morphogenesis. MDCK cells transfected with either a single siRNA or a pool of JAM-A-specific siRNAs were grown in a three-dimensional (3D) collagen matrix to develop cysts. Spindle orientation in mitotic cells was analysed using confocal microscopy as described before^17^ (see also Methods and Fig. 1b for a schematic illustration of spindle analysis). In control cysts, the majority of spindles are oriented perpendicular to the centroid of the cyst (mean angles: 73±2° (mean±s.e.m.) for single siRNA, 70±1° for siRNA pool, *n*=3; Fig. 1c–e), which is similar to what has been observed in Caco-2 epithelial cells^17^. In JAM-A knockdown cells, spindle orientation was almost randomized (mean angles=57±3° for single siRNA, 53±6° for siRNA pool, *n*=3; Fig. 1c–e). Ectopic expression of mouse JAM-A, which is not targeted by the canine JAM-A-specific siRNAs, completely restored spindle orientation (71±3° in the absence of cJAM-A short hairpin RNA (shRNA), 49±5° in the presence of cJAM-A shRNA, 75±1° in the presence of cJAM-A shRNA and mJAM-A complementary DNA, *n*=3; Fig. 1d,e and Supplementary Fig. 1). Previous studies had shown that the mitotic spindle apparatus positions the apical membrane at the center of the developing cyst to regulate the development of a single lumen during cyst morphogenesis^17^. When we analysed lumen formation, we found that JAM-A knockdown resulted in the formation of cysts with multiple lumens, without affecting apico-basal membrane polarity (Fig. 2). Ectopic expression of shRNA-resistant mJAM-A restored single lumen formation in JAM-A knockdown cells (Fig. 2). These results indicated that JAM-A is required for the planar orientation of the mitotic spindle in polarized epithelial cells. To further corroborate these findings, we ectopically expressed a mutant of JAM-A lacking the first Ig-like domain (ΔV-JAM-A), which is dimerization deficient and acts in a dominant-negative manner in TJ formation and epithelial cell migration^15^,^18^. Expression of ΔV-JAM-A resulted in a significant randomization of the spindle angle (mean angle=50±5° versus 67±2° in control cells, *n*=3; Supplementary Fig. 2B,C) and in multilumenal cyst formation (Supplementary Fig. 2D). Taken together, these observations indicate that JAM-A regulates planar spindle orientation during epithelial morphogenesis.

### Regulation of mitotic Cdc42 activity by JAM-A

**Figure 3:**
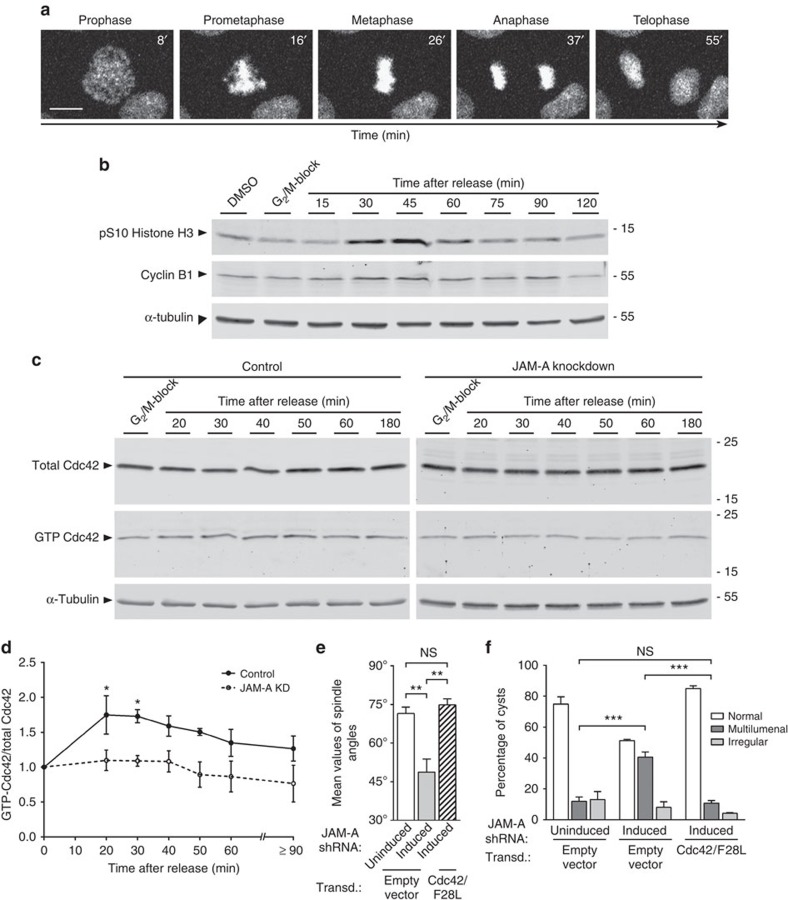
JAM-A controls Cdc42 activity during mitosis. (**a**) Mitosis in MDCK cells. MDCK cells were stained with Hoechst 33342 and observed under a fluorescence microscope. Images representative for distinct stages of mitosis were taken at the indicated time points. Mitosis was typically completed within 60 min after the first signs of nuclear condensation. Size bar, 5 μm. (**b**) RO-3306-treated cells proceed normally through mitosis after release from the RO-3306 block. MDCK cells were treated with RO-3306 as described in the Methods section. Cells were harvested at the indicated time points and analysed for the levels of two markers of mitosis, Ser10-phosphorylated histone H3 and Cyclin B1. (**c**) JAM-A activates Cdc42 during mitosis. MDCK cells stably transfected with a doxycycline-regulated JAM-A shRNA expression vector were either left untreated (control) or were treated with doxycycline to induce JAM-A shRNA expression (JAM-A knockdown), then synchronized with RO-3306 to arrest cells at G_2_/M-phase transition (G_2_/M block) and analysed for Cdc42 activity at the indicated time points after release from the G_2_/M block. (**d**) Quantification of Cdc42 activity of five independent experiments. Levels of active Cdc42 (GTP-Cdc42) were normalized to total Cdc42 levels and are expressed as signal intensities relative to the normalized GTP-Cdc42 levels of cells that were not released into mitosis (G_2_/M block). *P<0.05. (**e**,**f**) Cdc42/F28L restores planar spindle orientation and single lumen specification in JAM-A knockdown cells. MDCK cells with doxycycline-inducible JAM-A knockdown were transduced with lentiviral vectors expressing Cdc42/F28L. Cells were grown to cysts in 3D collagen gels. Spindle orientation (**e**) and lumen formation (**f**) were analysed in three independent experiments as described in Figs 1 and 2, respectively. Statistical analysis was performed using two-way repeated-measures ANOVA with Bonferroni's *post-hoc* test (**d**) or one-way ANOVA with Tukey's *post-hoc* test (**e**,**f**). The rescue experiments using Cdc42/F28L to restore mitotic spindle orientation (**e**) and single lumen specification (**f**) were performed in parallel with the rescue experiments using mJAM-A (shown in Figs 1e and 2b, respectively); the control samples are therefore identical. Data are expressed as means±s.e.m.; ns, not significant; *P<0.05, **P<0.01, ***P<0.001.

Rho family GTPases control cell morphogenesis by organizing the actin cytoskeleton and by regulating MT alignment dynamics^19^. During mitosis, Cdc42 but not Rac1 is activated by Ect2 and MgcRacGAP^20^,^21^. Cdc42 regulates spindle orientation in various cell types including polarized Caco-2 and MDCK epithelial cells^10^,^17^,^22^,^23^. We first analysed mitosis in polarized MDCK cells by live-cell imaging and found that MDCK cells complete mitosis within ∼60 min (Fig. 3a). To test whether Cdc42 acts downstream of JAM-A in the regulation of spindle orientation, MDCK cells were synchronized to G_2_/M phase transition using the Cdk1 inhibitor RO-3306, which blocks cell cycle progression at G_2_/M transition^24^. Treatment of MDCK cells with RO-3306 typically resulted in ∼80% of the cells with a DNA content of 4C (Supplementary Fig. 3A). After washout of RO-3306, cells proceeded normally through mitosis as indicated by histone H3 Ser10 phosphorylation and cyclin B1 levels, which both peaked at 30–45 min after release from the RO-3306 inhibition (Fig. 3b and Supplementary Fig. 3B)^25^,^26^. When we analysed the activity of Cdc42 in MDCK cells, we found that it transiently increased during mitosis, reaching peak levels at 20–30 min after release from the G_2_/M block (Fig. 3c,d), which corresponds to prometaphase–metaphase (Fig. 3a). This is similar to what has been observed in mitotic HeLa cells^21^. In JAM-A knockdown cells, the activity of Cdc42 did not increase during prometaphase and metaphase, and remained significantly below the levels of control cells (Fig. 3c,d), indicating that JAM-A activates Cdc42 during early phases of mitosis. To test whether JAM-A and Cdc42 operate in the same pathway of mitotic spindle orientation, we transfected JAM-A knockdown cells with a fast-cycling, self-activating mutant of Cdc42 (Cdc42/F28L). Ectopic expression of Cdc42/F28L completely restored planar spindle orientation (Fig. 3e) and single lumen specification (Fig. 3f) in JAM-A knockdown cells, indicating that Cdc42 acts downstream of JAM-A during spindle orientation. Together, these findings indicate that JAM-A operates upstream of Cdc42 to activate a signalling pathway, which regulates planar spindle orientation in polarized epithelial cells.

### JAM-A regulates the cortical localization of dynactin

**Figure 4:**
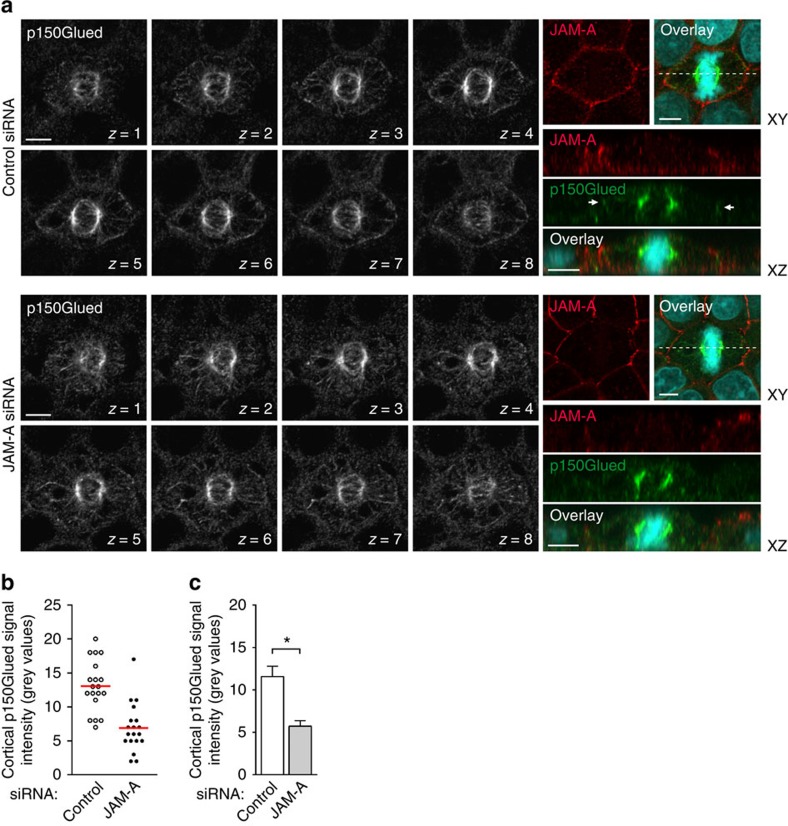
JAM-A controls cortical dynactin localization during mitosis. (**a**) *Z*-axial distribution of p150Glued. MDCK cells were transfected with either control siRNAs (top) or canine JAM-A-specific siRNAs (bottom). Optical sections (*Z*=1 to *Z*=8) were taken at 0.36-μm intervals. Right panels: representative examples of mitotic control cells (top) and JAM-A knockdown cells (bottom). Dotted lines in the *XY* panels indicate the positions of *XZ* sections shown underneath the *XY* panels. Small arrows in *XZ* panels point to cortical p150Glued (control siRNA panels only). Size bars, 5 μm. (**b**) Representative scatter diagram showing cortical p150Glued fluorescence intensity in control cells and JAM-A knockdown cells. (**c**) Quantitative analysis of cortical p150Glued fluorescence. Statistical analysis was performed with unpaired Student's *t*-tests. Data are presented as means±s.e.m. from three independent experiments. *P<0.05.

The stable attachment of astral MTs at the cortex is mediated by the dynein–dynactin complex, which simultanously interacts with the MT plus ends and with sites at the lateral cortex^3^,^27^. We therefore investigated whether JAM-A regulates the cortical localization of the dynein–dynactin complex in polarized epithelial cells. To this, we stained MDCK cells for p150Glued, a subunit of the dynactin complex^28^. In control metaphase cells, p150Glued was localized at the centrosomes, along spindle and astral MTs, as well as at the cell cortex where it appeared in cortical spots (Fig. 4a)^29^. In JAM-A knockdown cells, the cortical localization of p150Glued was reduced and the total fluorescence intensity along the entire lateral cortex reached ∼50% of the fluorescence intensity in control cells (Fig. 4a–c). These observations suggest that JAM-A is required for the stable localization of dynactin at the lateral cell cortex during mitosis.

### JAM-A regulates PI(3)K activity during mitosis

**Figure 5:**
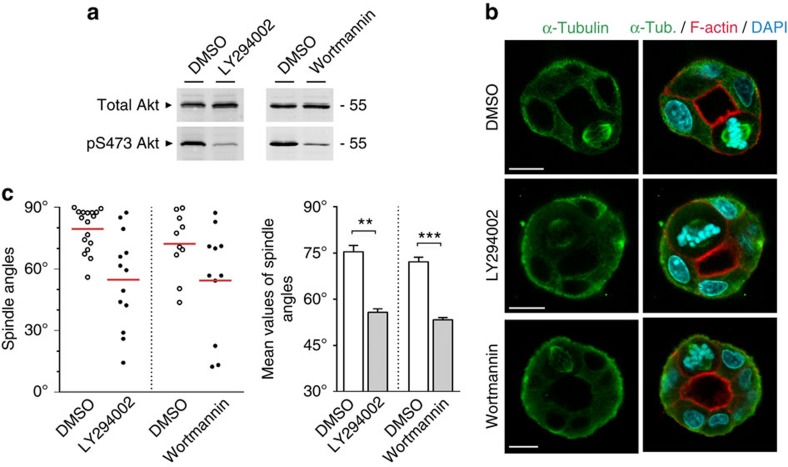
PI(3)K activity is required for planar spindle orientation in polarized epithelial cells. (**a**) Levels of PI(3)K activity in MDCK cells after treatment with PI(3)K inhibitors (LY294002, 100 μM, 2 h) and Wortmannin (100 nM, 1 h), as analysed by Ser473 phosphorylation of Akt. (**b**) Inhibition of PI(3)K activity disrupts planar spindle orientation. MDCK cells were grown in 3D collagen gels and treated with PI(3)K inhibitors LY294002 (100 μM, 2 h) or Wortmannin (100 nM, 1 h). Cysts were stained for α-tubulin (green), F-actin (red) and DNA (blue). Size bars, 10 μm. (**c**) Quantitative analysis of spindle orientation in LY294002- or Wortmannin-treated cells. Left panel: scatter diagram (single experiment) of spindle orientation in MDCK cells treated with LY294002 or Wortmannin. Right panel: quantitative analysis of spindle orientation in LY294002- or Wortmannin-treated cells. Statistical analyses were performed with unpaired Student's *t*-tests. Data are presented as means±s.e.m. from three independent experiments. **P<0.01, ***P<0.001.

**Figure 6:**
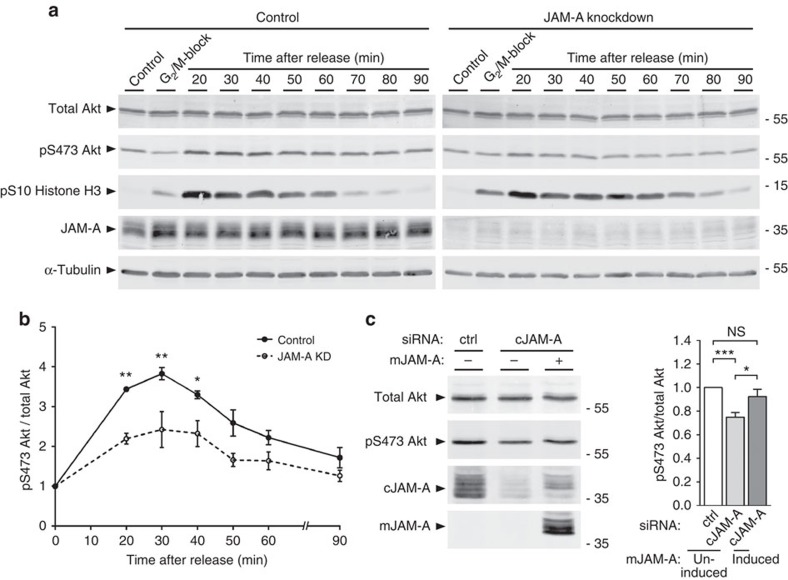
JAM-A regulates PI(3)K activation during mitosis. (**a**) MDCK cells expressing JAM-A shRNAs under a doxycycline-regulated promoter were synchronized at G_2_/M-phase transition, released into mitosis and analysed for PI(3)K activity using a phospho-Ser473-Akt-specific antibody. Progression through mitosis was monitored by analysing histone H3 phosphorylation at Ser10. Levels of JAM-A and α-tubulin in control cells and JAM-A knockdown cells were analysed by immunoblotting. (**b**) Quantification of PI(3)K activities. Levels of S473-phosphorylated Akt were normalized to total Akt levels and are expressed as signal intensities relative to the S473-phosphorylated Akt levels of cells that were not released into mitosis (G_2_/M block). (**c**) Ectopic expression of mouse JAM-A restores PI(3)K activity in JAM-A-depleted MDCK cells. Left panel: MDCK cells expressing murine JAM-A (mJAM-A) under a doxycycline-regulated promoter were transfected with siRNAs directed against endogenous canine JAM-A (cJAM-A). PI(3)K activity was analysed as in **a**. Right panel: PI(3)K activities 30 min after release from G_2_/M block in JAM-A KD cells in the absence or the presence of ectopic murine JAM-A relative to control conditions. Statistical analysis was performed using either two-way repeated-measures ANOVA with Bonferroni's *post-hoc* test (**b**, three independent experiments) or one-way repeated-measures ANOVA with Tukey's *post-hoc* test (**c**, ten independent experiments). Data are expressed as means±s.e.m.; ns, not significant; *P<0.05, **P<0.01, ***P<0.001.

In non-polarizing HeLa cells, the cortical accumulation of dynactin depends on the activity of the lipid kinase PI(3)K, which acts downstream of Cdc42 (ref. 10). We first addressed the question whether PI(3)K is involved in spindle orientation in polarized epithelial cells. MDCK cells were grown in 3D culture, which promotes the development of apico-basal polarity^30^, in the presence of PI(3)K inhibitors. Inhibition of PI(3)K with either LY294002 or Wortmannin resulted in randomization of the mitotic spindle angles (mean angles: 55±6° for LY294002, 54±8° for Wortmannin, *n*=3; Fig. 5). These results indicate that PI(3)K activity is necessary for planar spindle orientation when cells develop full apico-basal polarity. To test whether JAM-A regulates the activity of PI(3)K during mitosis, we analysed PI(3)K activity in JAM-A-depleted mitotic cells. MDCK cells were synchronized to G_2_/M phase transition, then released into mitosis. At different time points after release from the G_2_/M block, PI(3)K activity was analysed by immunoblotting cell lysates with antibodies against Ser473-phosphorylated Akt. In control cells, PI(3)K activity increased during mitosis and reached peak levels between 20 and 30 min after release (Fig. 6a, b), which correlates with metaphase and which is similar to what is observed for Cdc42 (Fig. 3). In JAM-A knockdown cells, PI(3)K activity remained significantly below the levels observed in control cells for the first 40 min of mitosis (Fig. 6a,b). Ectopic expression of mouse JAM-A restored PI(3)K activity in JAM-A knockdown cells (Fig. 6c). These observations thus indicate that PI(3)K is transiently activated during early phases of mitosis in polarized epithelial cells, and that JAM-A is required for this transient activation.

### JAM-A regulates cortical PtdIns(3,4,5)P3 gradient formation

To test whether JAM-A-mediated activation of PI(3)K during mitosis is required to generate a PtdIns(3,4,5)P3 gradient at the cell cortex, we analysed PtdIns(3,4,5)P3 localization using the pleckstrin homology (PH) domain of Akt fused to green fluorescent protein (GFP) (Akt-PH-GFP), which was previously used to detect PtdIns(3,4,5)P3 at the cortex of dividing HeLa cells^9^. The Akt-PH-GFP biosensor localized specifically at the lateral cell–cell contacts of MDCK cells and this localization was disturbed after incubation with LY294002, confirming its specificity for PtdIns(3,4,5)P3 (Supplementary Fig. 4).

**Figure 7:**
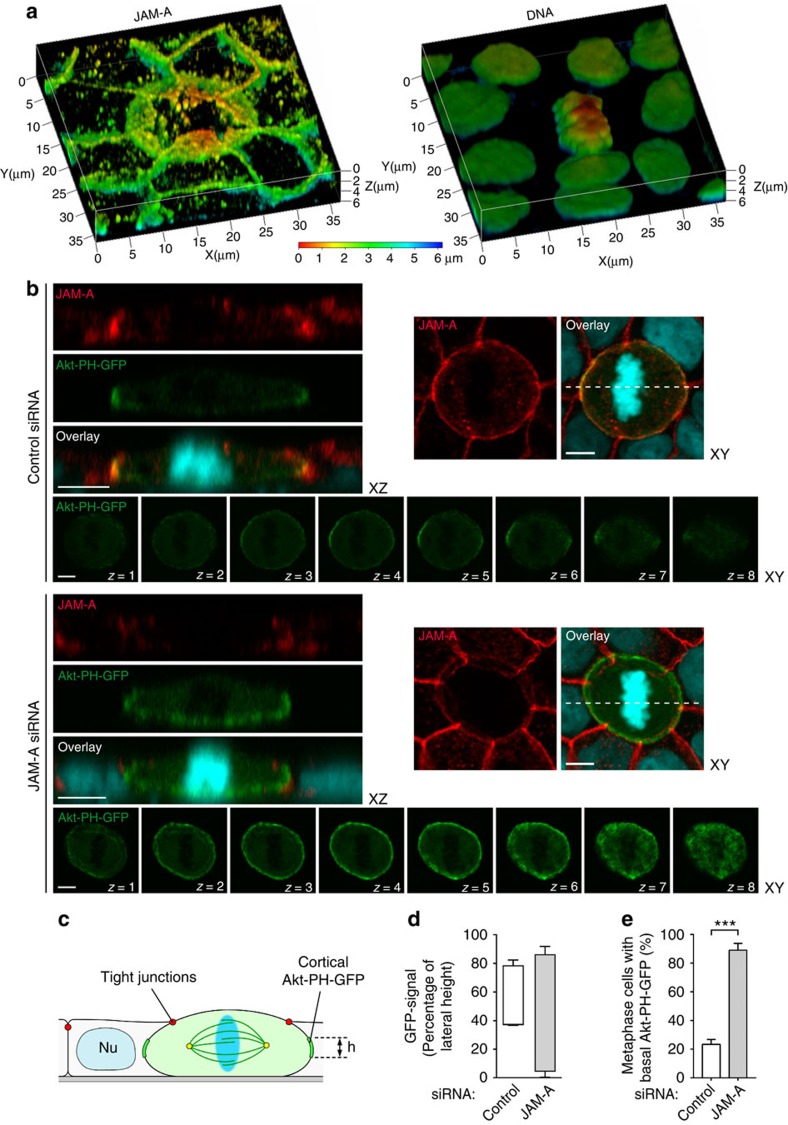
JAM-A controls cortical PtdIns(3,4,5)P3 localization during mitosis. (**a**) MDCK cells round up during mitosis. 3D view of a mitotic MDCK cell stained for JAM-A (left) and DNA (right). The heat map represents depth coding and indicates relative positions from the apex (red) to the basal side (blue) of the cells. Mitotic MDCK cells round up and are extensively overlapped by neighbouring cells. (**b**–**e**) Knockdown of JAM-A results in mislocalization of PtdIns(3,4,5)P3. (**b**) Distribution of Akt-PH-GFP in JAM-A knockdown cells. MDCK cells stably expressing Akt-PH-GFP were transfected with control siRNAs (top set of panels) or JAM-A siRNAs (bottom set of panels) and mixed with wild-type MDCK cells. The localization of JAM-A (red), Akt-PH-GFP (green) and DNA (blue) was analysed by fluorescence microscopy. To visualize the distribution of Akt-PH-GFP along the *XY* axis, optical sections (*Z*=1 to *Z*=8) were taken at 0.36-μm intervals. Right panels: representative examples of mitotic control cells and JAM-A knockdown cells. Dotted lines indicate the positions of *XZ* sections shown in the left panels. It is noteworthy that in the JAM-A siRNA sample only the cell in the centre is depleted for JAM-A. Size bars, 5 μm. (**c**) Schematic illustration of cortical Akt-PH-GFP analysis using the TJs as apical and the sites of cell–matrix interaction as basal boundary of the lateral membrane domain. (**d**) Distribution of Akt-PH-GFP along the lateral membrane domain in control cells and JAM-A knockdown cells. (**e**) Quantitative analysis of metaphase cells with basal localization of Akt-PH-GFP. Statistical analyses were performed with unpaired Student's *t*-tests. Data are presented as means±s.e.m. from three independent experiments (**d**,**e**). ***P<0.001.

To analyse the role of JAM-A in the localization of PtdIns(3,4,5)P3, MDCK cells were grown on polycarbonate filters to allow full polarization and the cortical distribution of the Akt-PH-GFP signal along the *Z* axis of mitotic cells was analysed by confocal microscopy. Mitotic MDCK cells rounded up and were overlapped by adjacent interphase cells, both at the apical and the basal side (Fig. 7a), as observed before^31^. JAM-A co-localized with occludin at the TJs but also with β-catenin along the lateral cortex below the TJs (Supplementary Fig. 5). In control MDCK cells, the Akt-PH-GFP fluorescence signal co-localized with JAM-A at cortical areas in projections from the spindle axis (Fig. 7b) where it covered ∼40% (41±5%, *n*=3) of the lateral cell height (Fig. 7d). In JAM-A siRNA-transfected cells, the Akt-PH-GFP fluorescence was distributed along the entire cell perimeter and, more importantly, it was not restricted to the midcortical area but was distributed along the entire basolateral membrane domain and covered ∼80% (81±4%, *n*=3) of the lateral cortex (Fig. 7b,d). The majority of JAM-A-depleted cells (89±5 versus 23±3% in control cells, *n*=3) showed mislocalization of the Akt-PH-GFP signal to the basal membrane domain (Fig. 7e). Together, these observations indicate that JAM-A regulates the midcortical accumulation of PtdIns(3,4,5)P3 at cell–cell contacts during mitosis.

### JAM-A regulates the cortical F-actin organization

**Figure 8:**
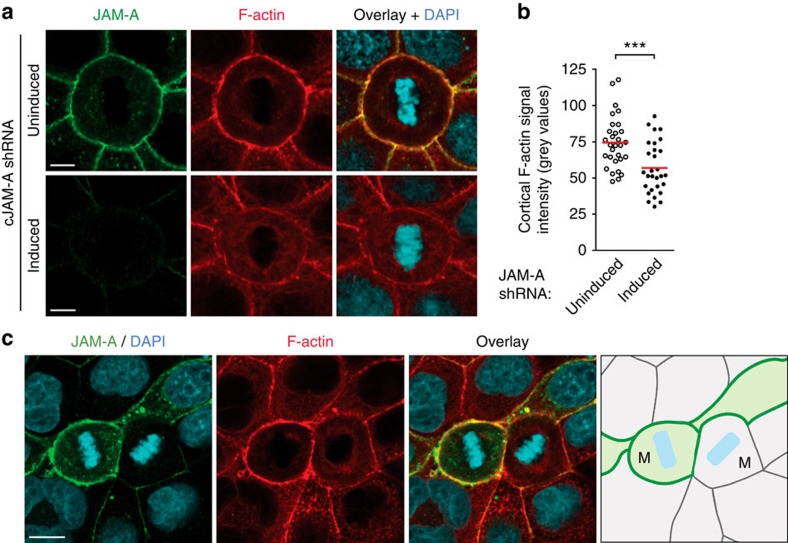
JAM-A regulates the formation of a cortical actin cytoskeleton during mitosis. (**a**) MDCK cells stably transfected with a doxycycline-regulated JAM-A shRNA were stained for JAM-A (green), F-actin (red) and DNA (blue) as indicated. Scale bars, 5 μm. (**b**) Scatter diagram showing cortical TRITC-phalloidin fluorescence intensity in control cells (uninduced) and JAM-A knockdown cells (induced). Statistical analysis was performed with unpaired Student's *t*-test. Sample numbers are *n*=30 for uninduced cells and *n*=29 for induced cells. ***P<0.001. (**c**) Midcortical section of two contacting metaphase cells, one without JAM-A knockdown (left) and one with JAM-A knockdown (right). The image shows a few cells that failed to respond to the doxyxcycline treatment (green cells in the schematic view) surrounded by JAM-A-depleted cells (grey cells in the schematic view). It is noteworthy that the cortical TRITC-phalloidin fluorescence intensity is strongly reduced in the JAM-A knockdown mitotic cell (right mitotic cell) when compared with the JAM-A-expressing mitotic cell (left mitotic cell). M, mitotic cell. Scale bar, 10 μm.

Besides the activation of PI(3)K, Cdc42 regulates the formation of a cortical actin cytoskeleton^10^, which is required for the stable attachment of astral MTs at the cortex in various cell types including polarized epithelial cells, non-polarizing epithelial cells and fibroblasts^8^,^29^. As Cdc42 acts downstream of JAM-A during mitotic spindle orientation (Fig. 3e,f), we tested whether JAM-A is involved in the formation of the cortical actin cytoskeleton during mitosis. Mitotic MDCK cells with or without knockdown of JAM-A were stained with tetramethylrhodamine (TRITC)-phalloidin to visualize F-actin. Mitotic cells rounded up and showed increased TRITC-phalloidin intensity at cell–cell junctions as compared with surrounding interphase cells (Fig. 8a,c), consistent with the modulation of the cortical actin network density and the stiffening of the cortical actin cytoskeleton during mitosis^32^,^33^,^34^. In JAM-A knockdown metaphase cells, the F-actin signal intensities at the cortex were significantly weaker than in control cells (Fig. 8). In addition, some cells failed to round up but instead retained a rather polygonal cell shape (Fig. 8c), similar to interphase cells. These observations indicate that JAM-A is required for the formation of a cortical F-actin cytoskeleton during mitosis. Studies in HeLa cells indicate that the cortical actin cytoskeleton is required for the stable localization of dynein at the cortex, and that PtdIns(3,4,5)P3 regulates the midcortical accumulation of dynactin^9^,^10^. As JAM-A knockdown results in reduced cortical dynactin (Fig. 4), we tested which of the two pathways regulates dynactin localization in polarized epithelial cells. Inhibition of PI(3)K activity with LY294002 or Wortmannin did not remove dynactin from the cortex (Supplementary Fig. 6A). In contrast, inhibition of actin polymerization with Latrunculin B or Cytochalasin D strongly impaired the localization of dynactin at the cortex of mitotic cells (Supplementary Fig. 6B). These findings suggest that JAM-A regulates the localization of dynactin at the cortex through its activity on the cortical actin cytoskeleton.

## Discussion

To maintain the organization of the epithelial sheet and to prevent cells from migrating out of the cell layer, individual epithelial cells must develop stable cell–cell contacts with their neighbours and must divide in the plane of the sheet. An instructive role of cell–cell adhesion for planar cell division has been proposed many years ago^35^. However, despite the large number of cell adhesion molecules that were identified at cell–cell contacts and found to regulate cell–cell contact formation and integrity, the molecular nature of the cell adhesion molecules, which regulate planar spindle orientation, is poorly understood. In this study we identify JAM-A as a provider of a junctional signal that is required for planar spindle orientation during mitosis. JAM-A acts upstream of Cdc42 to trigger a signalling pathway, which generates a PtdIns(3,4,5)P3 gradient at the lateral cortex and which regulates the formation of a cortical F-actin cytoskeleton during mitosis. This activity of JAM-A is required for the stable localization of dynactin at the lateral cortex. The cortically immobilized dynein–dynactin motor protein complex is thought to regulate the positioning of the mitotic spindle through its minus end-directed motor protein activity and perhaps through its ability to remain associated with depolymerizing MTs^36^.

The only other adhesion molecules known so far to influence spindle orientation in polarized epithelial cells are E-cadherin and Cadherin-6 (ref. 37). Both seem to act redundantly in regulating the localization of adenomatous polyposis coli at cell–cell contacts of MDCK cells^37^. Adenomatous polyposis coli has been implicated in symmetric cell division in epithelial tissues^38^ and this function could be based on its ability to interact with the MT plus-end-binding protein EB1 (ref. 39). However, perturbing the function of E-cadherin or Cadherin-6 has no influence on the localization of NuMA and LGN, nor does it affect the localization of dynein^37^, indicating that cadherins influence mitotic spindle orientation through an as-yet unknown mechanism.

A major finding in this study is that JAM-A activates PI(3)K during mitosis, and that PI(3)K activity is required for planar spindle orientation in polarized epithelial cells. A role for PI(3)K activity for planar spindle orientation has been observed in non-polarizing adherent HeLa cells but was found to be not required in polarized MDCK cells^9^. The discrepancy between these earlier observations and our findings can most likely be explained by the use of uncoated glass cover slips as supports for cell growth in the mentioned study. MDCK cells grown on non-permeable supports do not fully polarize and do not reach normal cell heights, probably because of a lack of nutrient uptake through the basolateral domain^40^. In our studies, we have cultured MDCK cells in a 3D collagen matrix allowing basolateral uptake and apical transport of nutrients^40^. A lack of full polarization and the concomitant reduced cell height might constrain spindle rotation and mask the requirement of the PI(3)K–PtdIns(3,4,5,)P3 pathway in polarized epithelial cells. Consistent with this notion we found that the mean variation of spindle angles is much smaller when cells are grown on polycarbonate filters as compared with 3D collagen gels (Supplementary Fig. 2C,E).

Using the Akt PH domain fused to GFP (Akt-PH-GFP) as a sensor for PtdIns(3,4,5)P3 localization, we made two observations: first, in JAM-A-depleted cells the Akt-PH-GFP fluorescence was no longer restricted to cortical sites in the projection of the mitotic spindle but instead was distributed around the cell perimeter. The mechanisms that determine the orientation of the mitotic spindle along the *XY* axis are poorly understood. Interestingly, overexpression of LGN in MDCK cells results in oscillations of the mitotic apparatus in the plane of the cellular sheet as a result of unbalanced pulling forces exerted by the astral MTs^5^. We hypothesize that JAM-A might prevent oscillation of the mitotic apparatus by restricting PtdIns(3,4,5,)P3 localization to specific positions at the cell perimeter. Second, in the absence of JAM-A, Akt-PH-GFP is mislocalized along the entire basolateral membrane domain. How JAM-A depletion results in basal localization of Akt-PH-GFP rather than in reduced Akt-PH-GFP signal intensity is presently unclear. One possible explanation would be that JAM-A negatively regulates a phosphoinositide (PI) phosphatase that removes the phosphate residue from the 5-position of PtdIns(3,4,5)P3, thus generating PtdIns(3,4)P2, which is also recognized by the Akt-PH biosensor^41^. The most probable PI phosphatases are the Src homology 2 domain-containing inositol phosphate 5-phosphatase (SHIP) 1 and 2 (ref. 42). Interestingly, SHIP2 is localized at the basolateral membrane domain of MDCK cells^43^ and co-localizes with paxillin at focal contacts of HeLa cells^44^. The previously described correlation between JAM-A expression and β1 integrin levels^45^ could provide a link between JAM-A expression and SHIP2 localization, and/or activity at the basal membrane domain. As an alternative explanation for the increased Akt-PH-GFP signal intensity at the basal membrane domain in JAM-A knockdown cells, JAM-A could negatively regulate a specific PI(3)K isoform at the basal membrane domain of mitotic cells. Recently, the class I PI(3)K catalytic subunit p110δ has been found to be localized at the basal membrane domain of polarized MDCK cells where it controls apico-basal polarity and lumen formation^46^. The localization and activity of p110δ during mitosis has not been analysed and whether JAM-A negatively regulates the localization and/or activity of p110δ or a related isoform (p110α, p110β or p110γ) at the basal membrane domain during mitosis remains to be tested.

One major observation of our study is that JAM-A activates a signalling pathway to regulate the stable interaction of dynein with the cortex. This signalling pathway most likely bifurcates downstream of Cdc42 (ref. 10) and results in the generation of a PtdIns(3,4,5)P3 gradient at the lateral cortex and in the formation of a cortical actin cytoskeleton. As inhibition of PI(3)K activity using the two broad-spectrum PI(3)K inhibitors LY294002 and Wortmannin did not affect the stable localization of p150Glued at the cortex, whereas inhibition of actin polymerization with both Latrunculin B and Cytochalasin D prevented stable p150Glued localization at the cortex (Supplementary Fig. 6), we speculate that the immobilization of dynein at the cortex requires the actin-regulating activity of JAM-A, and that the activation of PI(3)K by JAM-A is required for the PtdIns(3,4,5)P3 gradient along the basolateral membrane domain.

The reorganization of the cortical actin cytoskeleton during mitosis probably serves several functions: immobilization of dynein after its offloading from the MTs^47^, maintenance of cell–cell contact integrity during cell rounding^31^ and generation of cortical rigidity to prevent MT plus ends from pulling membranes inwards and direct the pulling forces towards the centrosomes^48^. PtdIns(3,4,5)P3 localized at the midcortex could serve as physical anchor for proteins that link the cortex with the MT plus ends. Par3 would be a potential candidate to serve this function. Par3 can interact with dynein^49^ and with PIs including PtdIns(3,4,5)P3 (ref. 50) through non-overlapping domains, which would allow simultaneous binding of Par3 to both partners. Par3 has been found to control planar spindle orientation by regulating the formation of an apical Par6–aPKC complex that restricts LGN to the basolateral membrane domain^7^, but it is unknown whether Par3 at the basolateral membrane domain can link dynein to PtdIns(3,4,5)P3 during mitosis. Another potential candidate to link the cortex with the MT plus ends is NuMA. NuMA has recently been found to directly interact with the PIs PtdIns(4)P, PtdIns(4,5)P2 and PtdIns(3,4,5)P3 (refs 51, 52). However, this LGN/Gαi-independent interaction of NuMA with PIs regulates dynein localization during anaphase but not metaphase^51^,^52^. NuMA is therefore not a likely candidate to directly link dynein to PtdIns(3,4,5)P3 at the midcortex during metaphase. We analysed the cortical localization of NuMA in MDCK cells during metaphase and anaphase, and as described for HeLa cells and Cos7 cells^51^,^52^,^53^ we observed an increase in cortical NuMA during anaphase compared with metaphase (Supplementary Fig. 7). We observed no difference in cortical NuMA between control and JAM-A knockdown MDCK cells, neither during metaphase nor during anaphase (Supplementary Fig. 7), suggesting that JAM-A does not contribute to the cortical localization of NuMA.

Our study identifies JAM-A as cell adhesion receptor that provides cortical signals to regulate planar orientation of the mitotic spindle in polarized epithelial cells. JAM-A activates Cdc42, generates a cortical gradient of PtdIns(3,4,5)P3 and regulates the accumulation of F-actin at the cortex. Our study indicates that a signalling pathway that regulates planar spindle orientation in non-polarizing cells in the absence of cell–cell junctions depends on cortical cues in polarized epithelial cells embedded in a multicellular tissue. It will be important to address the question whether the NuMA–LGN–Gαi pathway also depends on junctional cues and, if so, which adhesion receptors provide these.

## Methods

### Cell culture and transfections

MDCK II wild-type cells (Sigma-Aldrich (SA), Munich, Germany) and MDCK II Tet-Off cells (BD Biosciences, Heidelberg, Germany) were grown in DMEM containing 10% FCS, 1% glutamine, 100 U ml^−1^ penicillin and 100 μg ml^−1^ streptomycin. MDCK II cells stably expressing the PH domain of Akt1 fused to GFP (Akt-PH-GFP) were generated by transfecting MDCK II cells with a construct containing AA 1–164 of human Akt1 fused to enhanced GFP in pEGFP-N1 (kindly provided by Dr Tamas Balla, NIH, Bethesda MD). Transfected cells were maintained in DMEM supplemented with 500 μg ml^−1^ G418. MDCK II Tet-Off cell lines stably expressing murine wild-type JAM-A or a murine JAM-A mutant lacking the membrane-distal V-type Ig-domain (ΔV-JAM-A) were maintained in DMEM medium supplemented with 100 μg ml^−1^ G418, 1 μg ml^−1^ puromycin, 150 μg ml^−1^ hygromycin and 50 ng ml^−1^ doxycycline. Expression of transgenes was induced by transferring cells into medium lacking doxycycline using tetracycline-free FCS (BD Biosciences)^15^. Downregulation of canine JAM-A (cJAM-A) in MDCK cells was performed by either transient transfection of cJAM-A siRNAs (5′-CCAGUAAGAAGGUGAUUUA-3′) using Lipofectamine 2000 (Invitrogen, Darmstadt, Germany) or by stable transfection with cJAM-A shRNA expression vectors. To generate cell lines stably expressing the cJAM-A shRNA under a tetracycline-regulated promoter, the siRNA was cloned as a double-stranded oligonucleotide into pEmU6pro-T (kindly provided by Dr Karl Matter, University College London). Stable MDCK II cell lines were generated by electroporation and subsequent selection by growth in media containing 500 μg ml^−1^ G418 and 5 μg ml^−1^ blasticidin^15^. In addition to single siRNAs, a pool of cJAM-A siRNAs (siTOOLs Biotech, Martinsried, Germany) was used for transient transfections. Rescue constructs encoding murine JAM-A (resistant against canine JAM-A-specific shRNA) or fast-cycling Cdc42 (Cdc42/F28L) were introduced into MDCK cells by lentiviral transduction. Lentiviral particles were produced in HEK293T cells by cotransfection of the lentiviral expression vector pCDH-CMV-MCS-EF1-Puro (System Biosciences, Mountain View, CA, USA) with the packaging vectors psPAX2 and pMD2.G (kindly provided by the laboratory of Professor Dr Didier Trono, Lausanne, Switzerland; Addgene plasmids 12260 and 12259, respectively) in a ratio of 4:3:1. Lentiviral particle-containing HEK293T supernatants were harvested 1 day after transfection. MDCK target cells were incubated with lentiviral supernatants for 24 h. Transduced cells were maintained in standard culture medium supplemented with 1 μg ml^−1^ puromycin.

### Antibodies and reagents

The following antibodies were used in this study: mouse mAb anti-p150Glued (BD Biosciences 610473, 1:500), mouse mAb anti-pan Akt (Cell Signaling Technology (CST), Frankfurt, Germany; 40D4, 2920, 1:2,000), rabbit mAb anti-pS473 Akt (CST D9E, 4060, 1:1,000), rabbit pAb anti-Cyclin B1 (CST B1, 4138, 1:200), mouse mAb anti-pS10 Histone H3 (CST 6G3, 9706, 1:1,000), rabbit pAb anti-NuMA (CST 3888, 1:500), rabbit pAb anti-canine JAM-A^15^, 1:500), rabbit pAb anti-aPKCζ (Santa Cruz, Heidelberg, Germany; C-20, sc-216, 1:200), rabbit pAb anti-Cdc42 (Santa Cruz P1, sc-87, 1:1,000), mouse mAb anti-Flag M2 (SA, F1804, 1:1,000), mouse mAb anti-α-tubulin (SA, clone B-5-1-2, T5168, 1:1,000), mouse mAb anti-γ-tubulin (SA, clone GTU-88, T6557, 1:1,000), mouse mAb anti-occludin (Zymed/Life Technologies, Rockford, IL, USA; 33-1500, 1:1,000), rabbit pAb anti-ZO-1 (Zymed 61-7300, 1:1,000). The fast-cycling mutant of hCdc42 (Cdc42/F28L) was generated by site-directed mutagenesis of hCdc42 in plasmid vector pGEX-2T (kindly provided by Dr Lars Helmsath, Max-Planck-Institute for Molecular Physiology, Dortmund) and subsequently subcloned into lentiviral vector pCDH-CMV-MCS-EF1-Puro. The following reagents were used in this study: Wortmannin (Applichem, Darmstadt, Germany), Cytochalasin D, Latrunculin B, LY294002 (Calbiochem/Merck, Darmstadt, Germany), RO-3306 (Enzo Life Sciences, Lörrach, Germany), Paclitaxel/Taxol and TRITC-phalloidin (SA).

### Cell synchronization

MDCK II cells were synchronized into mitosis with the cyclin-dependent kinase 1 inhibitor RO-3306, which arrests the cells at the G_2_/M-phase transition^24^. In brief, subconfluent cell populations were incubated for 10 h in regular growth medium containing 9 μM RO-3306 (G_2_/M-phase block). To release cells from the G_2_/M-block, cells were washed and grown in regular medium. To assess the rate of synchronization, cells were were fixed (2 h, ice-cold 70% ethanol) and incubated for 30 min in PBS containing 200 μg ml^−1^ DNase-free RNase A, 20 μg ml^−1^ propidium iodide, 0.1% Triton X-100. The DNA content of the cells was analysed by flow cytometry (FACSCalibur, BD Biosciences). Cell aggregates were discriminated by pulse processing and single cells were categorized based on to their DNA content as cell cycle phases G_1_/G_0_, S and G_2_/M.

### Cdc42 pull-down assays

Pull-down assays for active Cdc42 were performed using a method described in the literature^54^. Briefly, MDCK II cells were washed with ice-cold PBS and lysed with fish buffer (50 mM Tris/HCl pH 8.0, 150 mM NaCl, 1 mM CaCl_2_, 1 mM MgCl_2_, 1% Triton X-100) supplemented with a protease inhibitor cocktail (Roche). The crude lysates were immediately frozen in liquid N_2_ and stored at −80 °C. For complete lysis, the samples were thawed on ice and incubated for 10 min at 4 °C with constant agitation. Cell debris was removed by centrifugation (5 min at 17,000*g*, 4 °C) and total protein concentrations of the supernatants were determined by bicinchoninic acid assay. Equal amounts of total protein were incubated with 10 μg of recombinant GST-PAK-CRIB (plasmid kindly provided by Dr John Collard, The Netherland Cancer Institute, Amsterdam) coupled to glutathione Sepharose beads (GE Healthcare) for 30 min at 4 °C under continuous agitation. The beads were washed three times in fish buffer, bound proteins were eluted in Laemmli buffer and subjected to SDS–PAGE followed by western blot analysis with antibodies against Cdc42. The levels of total Cdc42 were analysed by immunblotting equal amounts of total lysates with anti-Cdc42 antibodies. Quantification of signal intensities was performed using the Odyssey imaging system (LI-COR Biosciences, Bad Homburg, Germany). For each band the integrated intensity (in K counts) was calculated with Odyssey application software (Version 3.0). The activity levels of Cdc42 were calculated by dividing the integrated intensity of GTP-Cdc42 signals by the integrated intensity of the corresponding total Cdc42 signals. Mean values and s.e. were calculated from five independent experiments. Statistical significance was evaluated using two-way repeated measures analysis of variance (ANOVA) with Bonferroni *post-hoc* test. *P*-values below 0.05 were considered as significant. Uncropped scans of all blots are shown in Supplementary Fig. 8.

### Immunofluorescence microscopy

For immunofluorescence microscopy, cells were grown on fibronectin-coated polycarbonate membrane filters (0.45 μm pore size, Corning, Amsterdam, The Netherlands). For all stainings, except NuMA and p150Glued, cells were washed with PBS containing Ca^2+^ and Mg^2+^, and fixed for 5 min with 4% paraformaldehyde (PFA). For NuMA or p150Glued stainings, cells were washed at 37 °C with PHEM buffer (300 mM PIPES/KOH pH 6.9, 125 mM HEPES, 50 mM EGTA, 20 mM MgCl_2_), treated for 1 min with warm (37 °C) PHEM buffer containing 0.5% Triton X-100 and 5 μM taxol, and fixed for 5 min either with −20 °C methanol or with 37 °C 4% PFA. PFA-fixed cells were permeabilized for 15 min in PBS containing 0.5% Triton X-100. After fixation, cells were washed with PBS containing 100 mM glycine, blocked for 1 h at room temperature (RT) (PBS, 10% FCS, 0.2% Triton X-100, 0.05% Tween 20, 0.02% BSA), then incubated with primary antibodies in blocking buffer for 1 h at RT or overnight at 4 °C. After incubation with the primary antibodies, cells were incubated with fluorochrome (AlexaFluor488, AlexaFluor568, AlexaFluor594 and AlexaFluor647)-conjugated, highly cross-adsorbed secondary antibodies (Invitrogen) for 2 h at RT. F-actin was stained with TRITC-phalloidin (SA), DNA was stained with 4,6-diamidino-2-phenylindole (SA) or DRAQ5 (Biostatus, Shepshed, UK). Samples were washed in blocking buffer and mounted in fluorescence mounting medium (Dako, Eching, Germany). Immunofluorescence microscopy was performed using the confocal microscopes LSM 510 Meta and LSM 780 (Carl Zeiss, Jena, Germany) equipped with the objective lenses Plan-Apochromat × 10/0.3, Plan-Neofluar × 20/0.5, Plan-Neofluar × 40/1.3 oil differential interference contrast, Plan-Apochromat × 63/1.4 oil differential interference contrast (Carl Zeiss). The distribution of fluorescence signals was analysed by taking optical sections of mitotic cells at 0.36-μm intervals. For the analysis of Akt-PH-GFP localization during mitosis, MDCK cells stably expressing Akt-PH-GFP were transfected with JAM-A-specific siRNAs and mixed with wild-type MDCK cells to avoid GFP fluorescence interference from neighbouring cells. The distribution of Akt-PH-GFP along the lateral membrane domain was analysed from the site of TJs and the basal membrane (see Fig. 7c for schematic illustration). Cortical fluorescence signals of p150Glued and F-actin at different optical sections were quantified using ImageJ software. Background fluorescence was subtracted from cortical fluorescence signals, resulting in normalized cortical fluorescence intensity.

### MDCK II cyst assays and 3D spindle-orientation analysis

MDCK II cells were seeded as single-cell suspension (3.4 × 10^4^ cells per ml) in 0.18% type I collagen from rat tail (BD Biosciences). After 3–6 days, the gels were washed in PBS, subjected to collagenase treatment (100 U ml^−1^ collagenase VII (SA), 15 min, RT) and fixed (4% PFA/PBS, 30 min, RT). Cells were permeabilized by treatment with 0.25% Triton X-100/PBS (30 min, RT) and washed extensively with 2% goat serum/PBS (1 h, RT). Incubations with primary and fluorochrome-conjugated secondary antibodies were performed in 2% goat serum/PBS for a minimum of 12 h at 4 °C. After extensive washing, the gels were mounted on glass coverslips using Kaiser's glycerol gelatine (Merck). Cysts were analysed using a confocal microscope as specified in the ‘Immunofluorescence microscopy' paragraph. To analyse mitotic spindle orientation, cysts grown for 2–3 days were fixed and stained for α-tubulin to visualize mitotic spindles, for F-actin or aPKCζ to visualize the apical membrane and for DNA. Confocal *Z*-stack images were taken at 1-μm intervals. The volumetric centre of the cysts was identified using ZEN 2010 software (Carl Zeiss). The spindle angle *α* was defined as the angle between the spindle axis and the line connecting the volumetric centre of the cyst with the centre of the spindle axis^17^ (see also Fig. 1b for a schematic illustration).

### Statistical analyses

Results are expressed as mean values±s.e.m. In experiments analysing lumen formation and spindle orientation, sample size required to detect large effects was estimated by power analysis using G*Power 3.1 (ref. 55). The D'Agostino and Pearson omnibus normality test was used to test the normality of data sample distributions. Comparisons of two groups were performed either with unpaired two-tailed Student's *t*-test or two-tailed Mann–Whitney test. Comparisons between two groups at different time points were performed using two-way repeated-measures ANOVA with Bonferroni *post-hoc* test. Comparisons of more than two groups were performed by one-way ANOVA or one-way repeated-measures ANOVA with Tukey's multiple comparisons test. Statistical analysis was performed using GraphPad Prism version 6 (GraphPad Software, San Diego, CA, USA). *P*-values are indicated as follows: *P<0.05, **P<0.01, ***P<0.001 and ****P<0.0001.

## Additional information

**How to cite this article:** Tuncay, H. *et al.* JAM-A regulates cortical dynein localization through Cdc42 to control planar spindle orientation during mitosis. *Nat. Commun.* 6:8128 doi: 10.1038/ncomms9128 (2015).

## Supplementary Material

Supplementary Information Supplementary Figures 1-8
