# Supplementary material for: JAM-A regulates cortical dynein localization through Cdc42 to control planar spindle orientation during mitosis
Source: Nat Commun. 2015 Aug 26;6:8128. doi: 10.1038/ncomms9128 (PMC4560831; doi:10.1038/ncomms9128)
Supplement: Supplementary Information — Supplementary Figures 1-8 [file ncomms9128-s1.pdf]

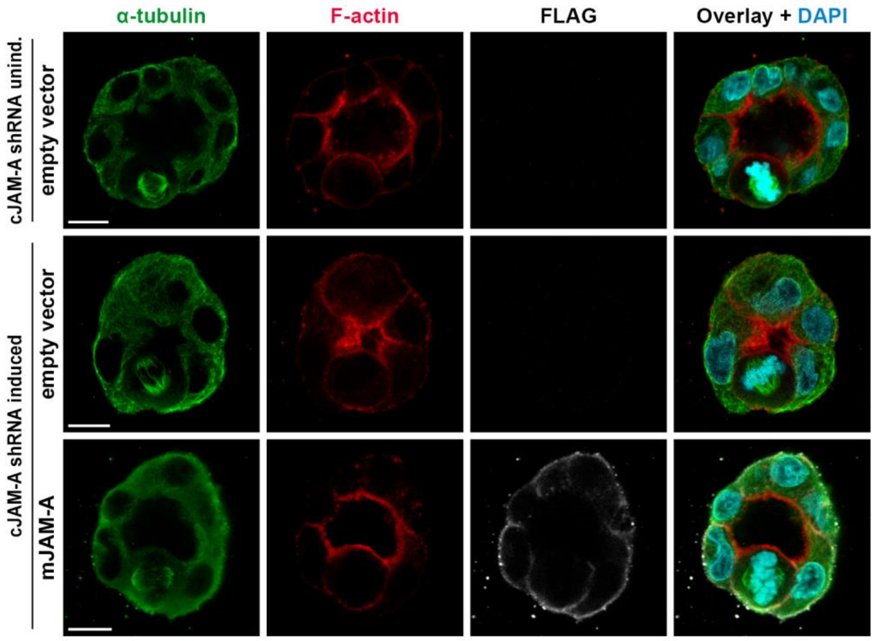

Supplementary Figure 1: Expression of shRNA-insensitive mouse JAM-A restores planar spindle orientation in JAM-A knockdown cells. MDCK cells stably expressing a canine JAM-A-specific shRNA under the control of a doxycycline-regulated promoter were transduced with lentiviral vectors expressing shRNA-resistant murine Flag-JAM-A (mJAM-A). Cells were grown in 3D collagen gels and stained for α-tubulin (mitotic spindle, green), F-actin (apical membrane, red), Flag epitope (mouse JAM-A, white) and DNA (blue). Note that ectopic expression of mJAM-A restores planar spindle orientation in JAM-A knockdown cells. Quantitation and statistical analysis of this experiment is shown in Figs 1D and 1E of the main article. Size bars: 10 μm.

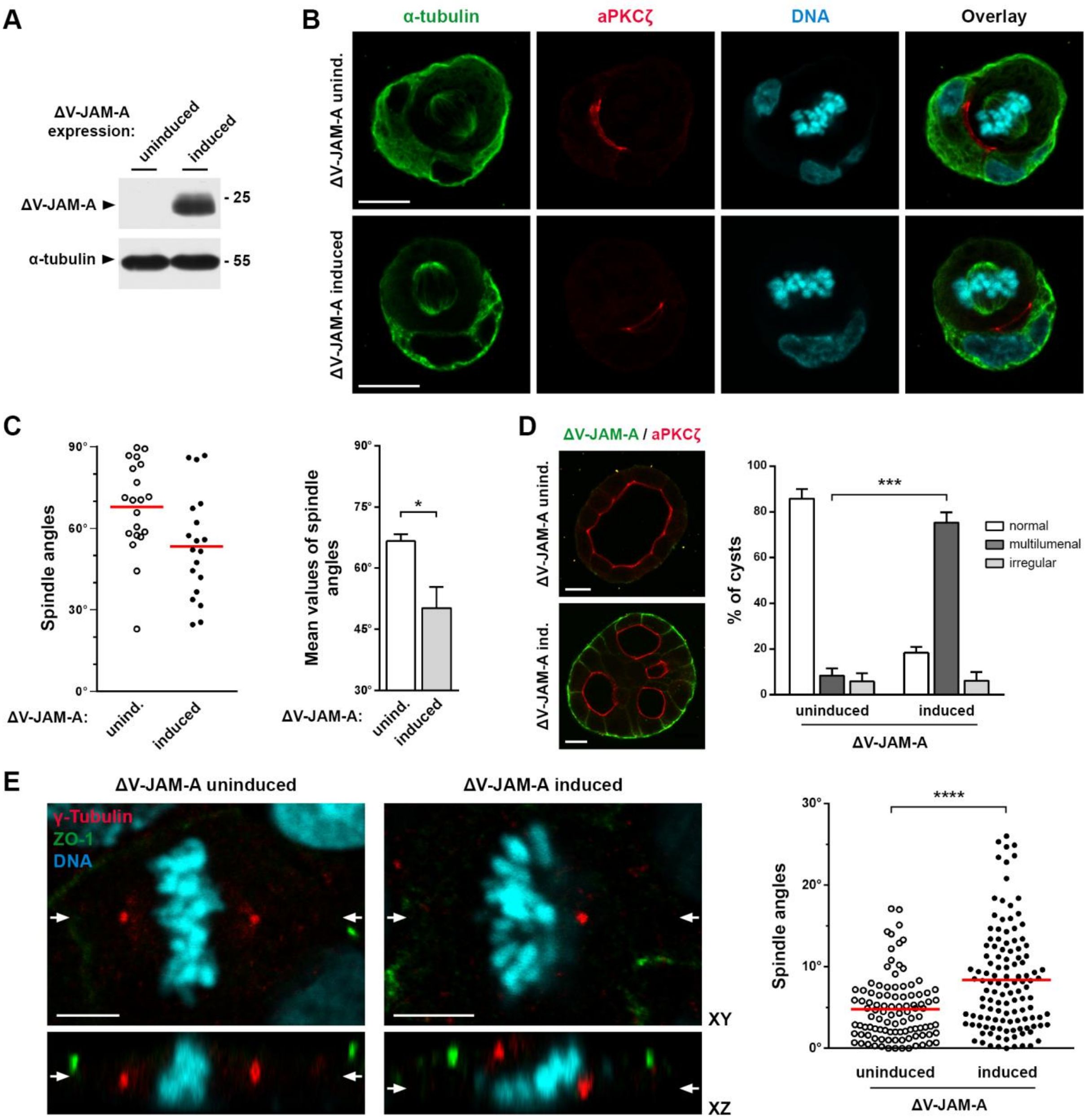

Supplementary Figure 2: Expression of dimerization-deficient ΔV-JAM-A results in aberrant spindle orientation and multilumenal cyst formation. (A) MDCK cells stably expressing ΔV-JAM-A under a doxycycline-regulated promoter were analyzed for the expression of ΔV-JAM-A by westernblot analysis. (B) ΔV-JAM-A MDCK cells were grown in 3D collagen gels and stained for α-tubulin (mitotic spindle, green), aPKCζ (apical membrane, red), and DNA (blue). Note that the mitotic spindle axis is misaligned in ΔV-JAM-A MDCK cells. Size bars: 10 µm. (C) Quantitation of spindle orientation in ΔV-JAM-A-expressing MDCK cells. Left panel: Representative scatter diagram of a single experiment before (uninduced) and after (induced) induction of ΔV-JAM-A expression. Right panel: Statistical analysis of spindle orientation in ΔV-JAM-A expressing MDCK cells before and after induction of ΔV-JAM-A expression. Statistical analysis was performed using unpaired Student's t-test with three independent experiments for each condition and is presented as means ± SEM. (D) Lumen formation in MDCK cysts upon expression of ΔV-JAM-A. Left panel: ΔV-JAM-A-transfected MDCK cells were left uninduced or were induced to express ΔV-JAM-A and grown for 6 - 8 days in 3D collagen gels to develop cysts. Cells were stained as indicated. Note that ΔV-JAM-A expression results in the formation of multiple lumens. Size bars: 10 µm. Right panel: Statistical analysis of lumen formation. Statistical analysis was performed using unpaired Student's t-test with three independent experiments in each condition and is presented as means ± SEM. Cysts were categorized as normal (white bar), multilumenal (dark bar), and irregular (grey bar). (E) Mitotic spindle orientation in ΔV-JAM-A-transfected MDCK cells grown in two dimensions. Left panels: MDCK cells were grown on polycarbonate filters to develop polarity, then fixed and stained for γ-tubulin to visualize the centrosomes and for ZO-1 to label the TJs; DNA was stained with Draq5. Arrows indicate the positions of sectional views. Top panels: positions of XZ-sectional views; bottom panels: positions of XY-sectional views. Size bars: 5 µm. Right panel: Scatter diagram showing spindle angles of MDCK cells before and after induction of ΔV-JAM-A expression. Mean values are indicated by a red bar. Statistical analysis was performed using two-tailed Mann-Whitney test. Sample numbers are $n = 101$ for uninduced and $n = 121$ for induced cells. *$p < 0.05$; ***$p < 0.001$; ****$p < 0.0001$.

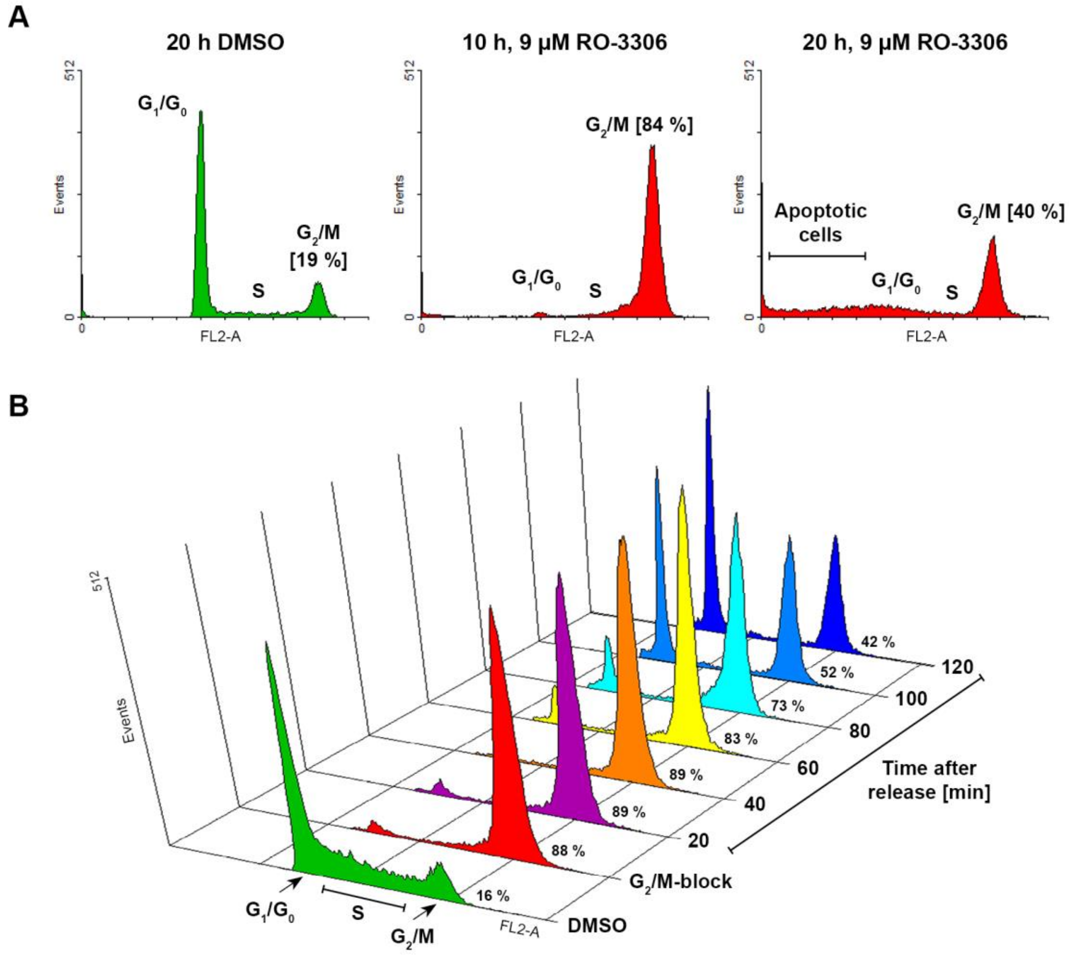

Supplementary Figure 3: Mitotic synchronization of MDCK cells with the Cdk1 inhibitor RO-3306. (A) Synchronization rates in RO-3306-treated MDCK cells. MDCK cells were incubated for the indicated time periods with solvent (DMSO) or with RO-3306 (9 µM). Cells were harvested, stained with propidium iodide, and analyzed by flow cytometry. The fluorescence intensities (X-axis) were plotted against the number of events (Y-axis). Treatment of cells with RO-3306 for 10 h typically resulted in approximately 80% of cells with 4C chromatin content (middle panel). Treatment for 20 h resulted in increased apoptotic cell death (right panel). (B) MDCK cells treated with RO-3306 proceed through mitosis and cytokinesis after wash-out of the inhibitor. MDCK cells were treated with RO-3306 (10 h, 9 µM), washed with PBS to remove the inhibitor, then further incubated without inhibitor for the indicated time periods. Vehicle-treated cells (DMSO) and cells that were not washed to remove the inhibitor (G_2/M block) served as controls. After 120 min, approx. 60% of the cells have completed cytokinesis.

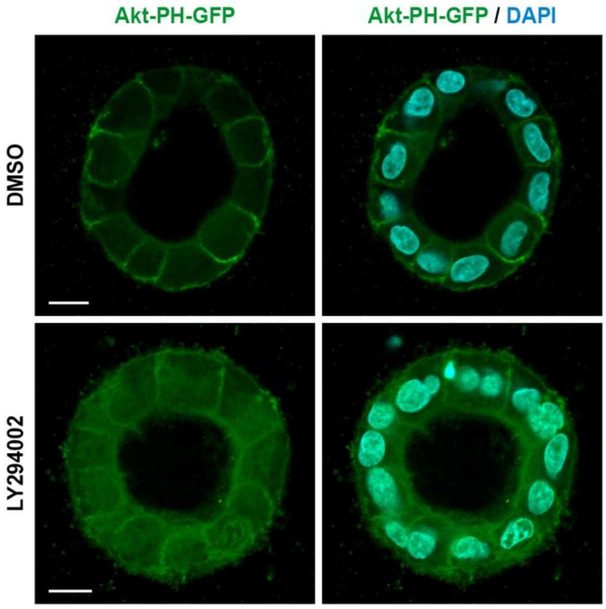

Supplementary Figure 4: Localization of Akt-PH-GFP in PI(3)K-inhibited cells. MDCK cells stably transfected with a cDNA encoding Akt-PH-GFP were grown in three-dimensional collagen gels. Cells were treated with either carrier (DMSO) or LY294002 (100 μM) for 2 h. Note that inhibition with LY294002 results in increased localization of Akt-PH-GFP in the cytoplasm. Size bars: 10 μm.

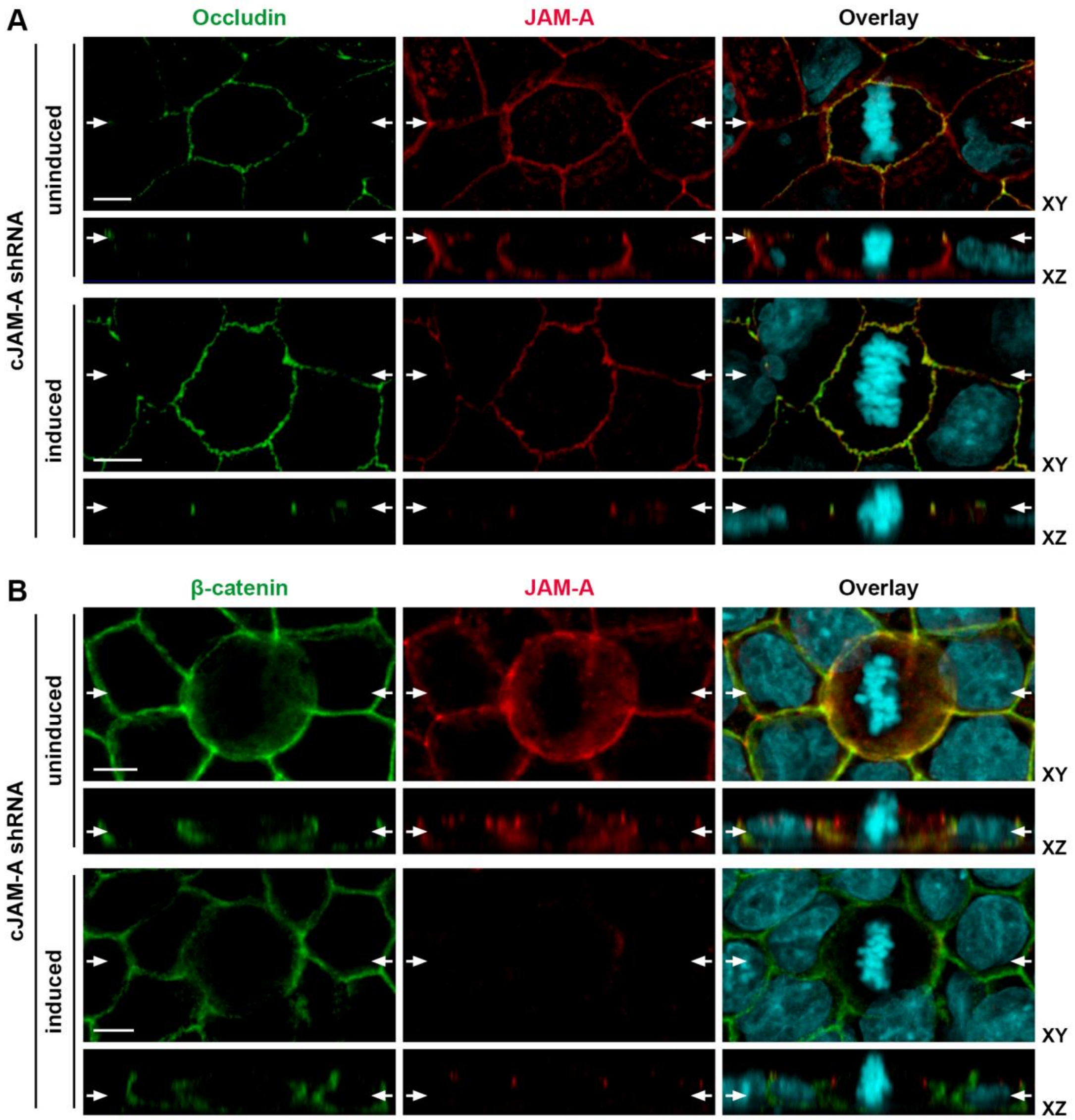

Supplementary Figure 5: JAM-A co-localizes with occludin and β-catenin in mitotic cells. (A) JAM-A co-localizes with occludin at TJs. MDCK II cells stably transfected with a tetracycline-regulated JAM-A shRNA-expression vector were left uninduced (top panels) or were incubated with doxycycline to induce JAM-A shRNA expression (bottom panel). Cells were stained with antibodies against occludin and JAM-A as indicated, DNA was stained with DAPI. JAM-A localizes along the entire lateral cortex and is enriched at the TJs. Note that the TJ-associated fraction of JAM-A molecules is still visible in JAM-A knockdown cells suggesting a slower turnover rate of TJ-associated JAM-A. Size bars: 5 µm. (B) JAM-A co-localizes with β-catenin during mitosis. MDCK cells were treated as described in (A) and stained with antibodies against β-catenin and JAM-A, DNA was stained using DAPI. Note that the major pool of JAM-A is localized along the lateral junctions and that this pool of JAM-A is completely depleted in JAM-A knockdown cells. Size bars: 5 µm. Arrows indicate the positions of the optical XZ and XY sections.

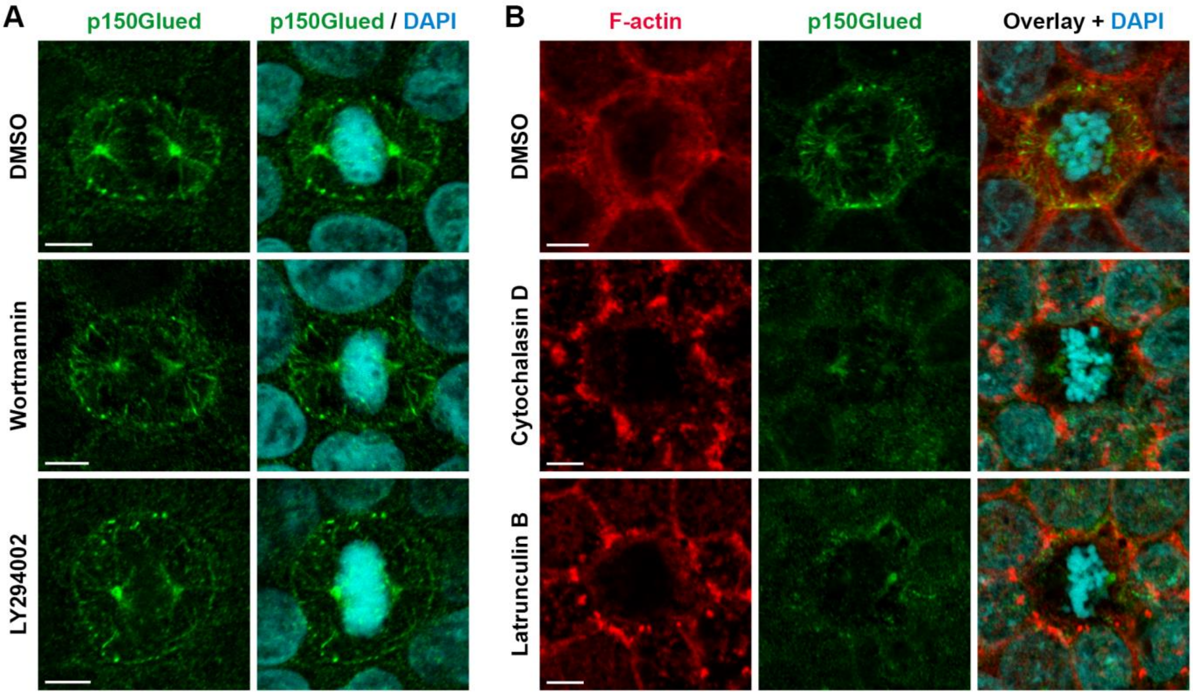

Supplementary Figure 6: PI(3)K and the F-actin cytoskeleton contribute differentially to the cortical localization of p150Glued. (A) Inhibition of PI(3)K does not prevent cortical p150Glued localization during mitosis. MDCK cells were incubated with the broad-specific PI(3)K inhibitors LY204002 (100 µM, 2 h) and Wortmannin (100 nM, 2 h). Cells were fixed and stained with antibodies against p150Glued and with DAPI to visualize DNA. (B) Inhibition of actin polymerization prevents cortical p150Glued localization during mitosis. MDCK cells were incubated with Latrunculin B (1 µM, 2 h) or Cytochalasin D (1 µg/ml 2 h). Cells were fixed and stained with antibodies against p150Glued and with DAPI to visualize DNA. Size bars: 5 µm.

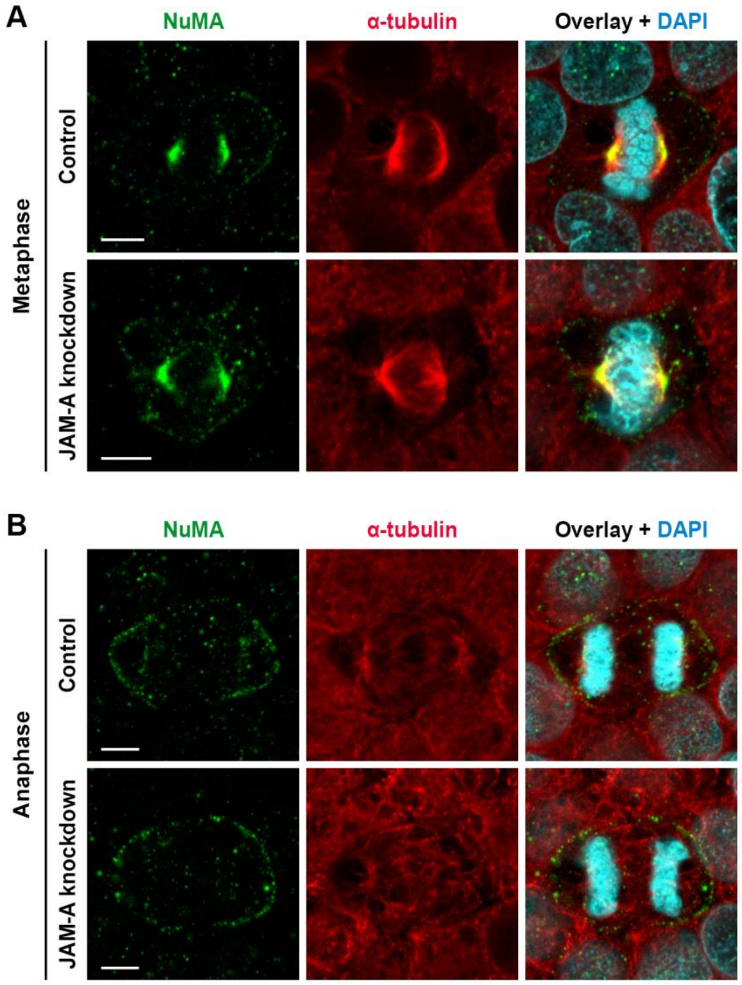

Supplementary Figure 7: The cortical localization of NuMA is unchanged in JAM-A knockdown cells. Localization of NuMA in JAM-A knockdown cells during metaphase (A) and anaphase (B). MDCK cells stably transfected with a tetracycline-regulated JAM-A shRNA-expression vector were left uninduced (top panels) or were induced with tetracycline to express JAM-A shRNA (lower panels). Cells were stained with antibodies against NuMA and against α-tubulin as indicated, DNA was stained with DAPI. Note that NuMA localization is not altered in JAM-A knockdown cells, neither during metaphase nor during anaphase. Size bars: 5 µm.

All western blots except Supplementary Figure 2A were scanned using a two channel infrared imager. The immunoblot in supplementary figure 2A was acquired by standard ECL reaction and film exposure.

▬ = Parallel detection of two fluorescence channels (680 and 800 nm) with infrared imager

▬ = Secondary detection after stripping the blot membrane

IIIIII = Membrane was cut to detect several proteins from the same immunoblot

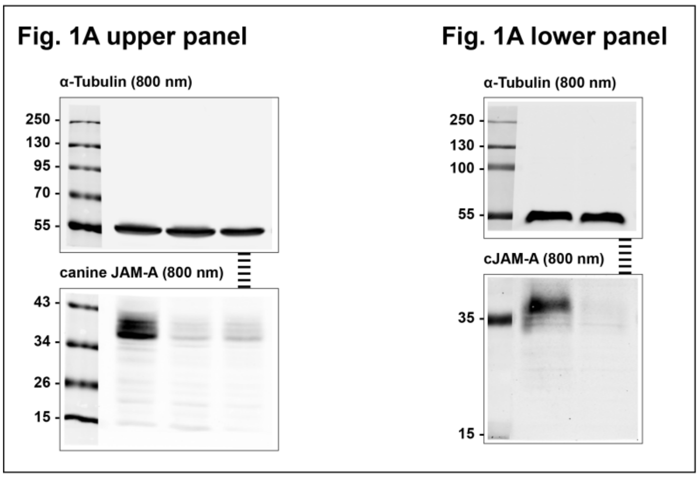

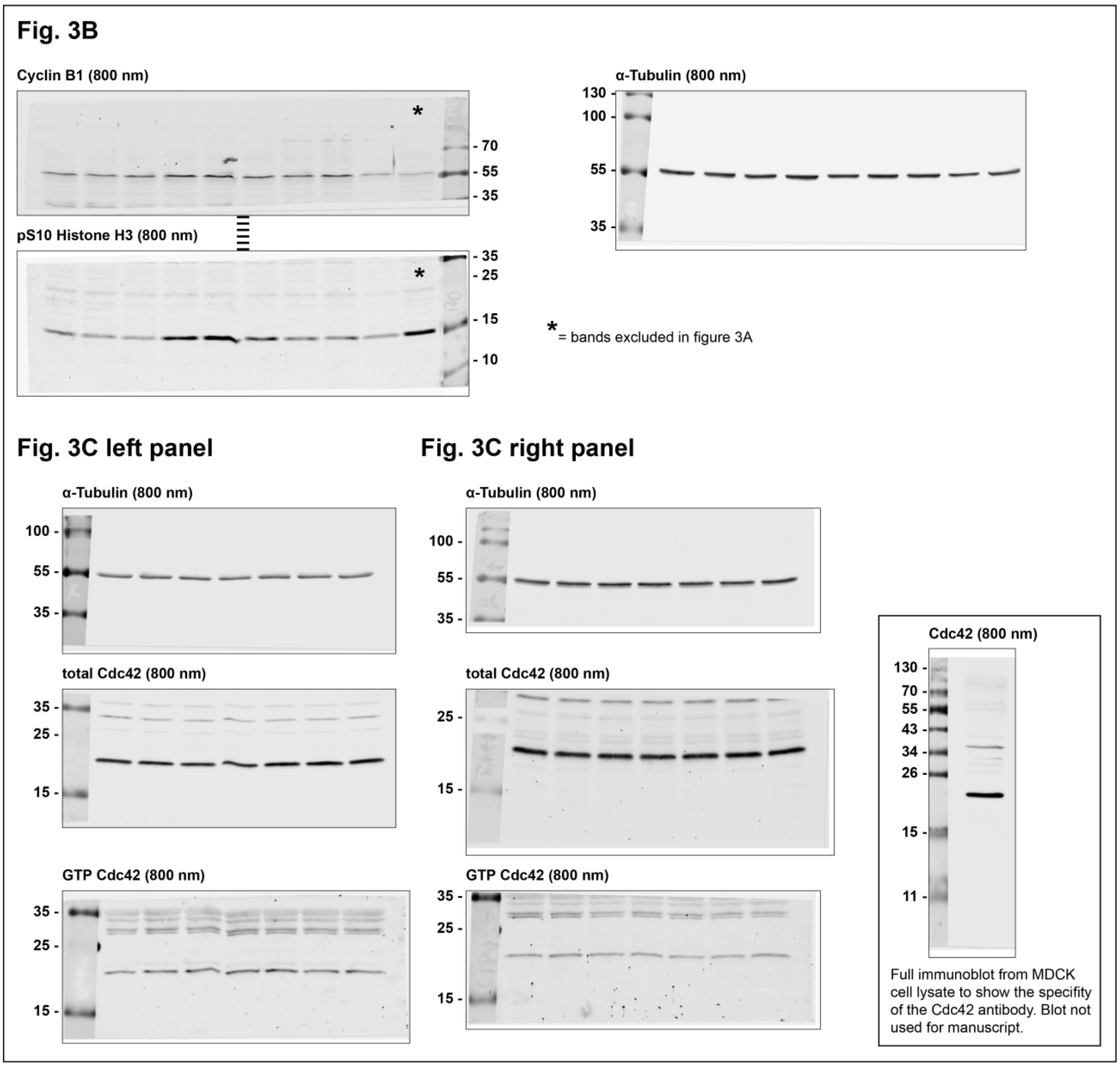

Supplementary Figure 8: Uncropped western blots of Fig. 1, 3, 5, 6, and Supplementary Fig. 2

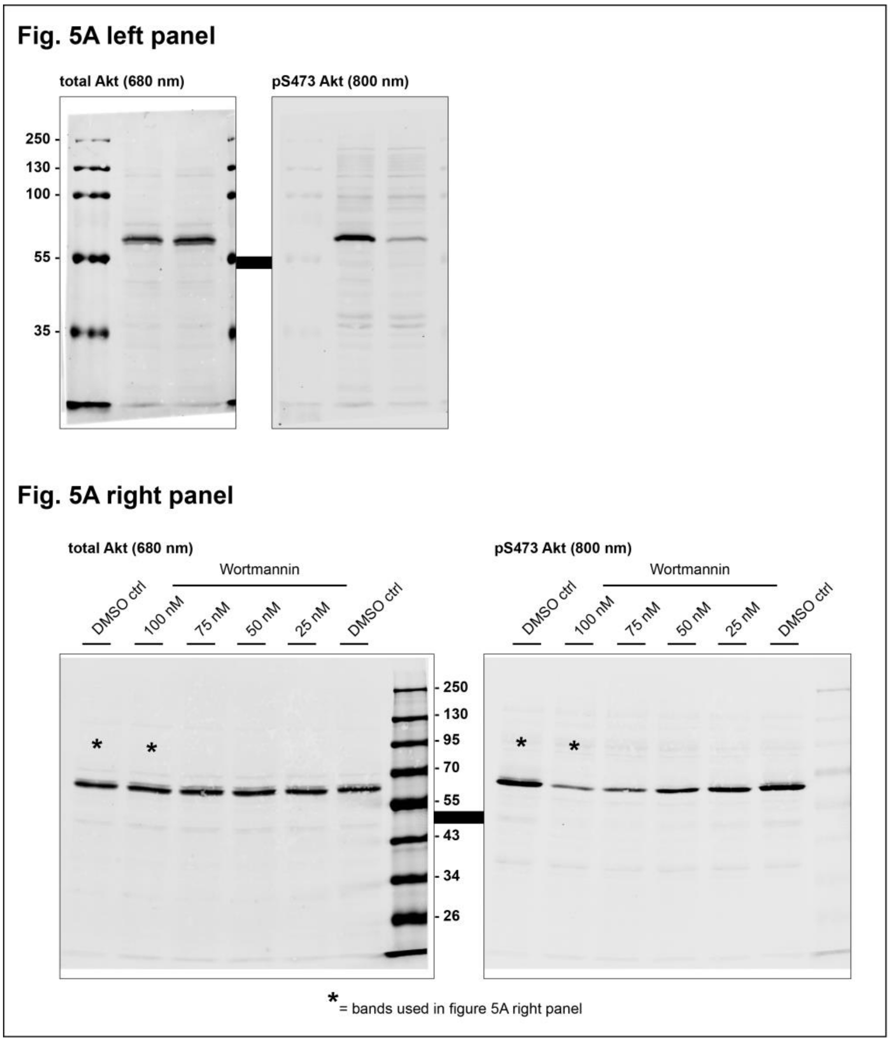

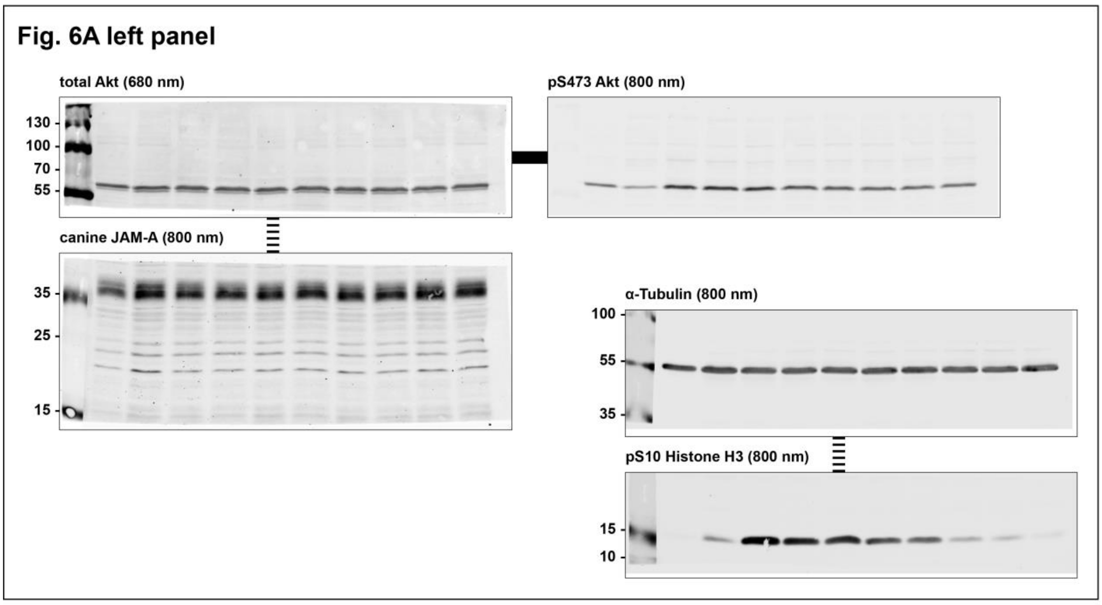

Supplementary Figure 8 continued

Fig. 6A right panel

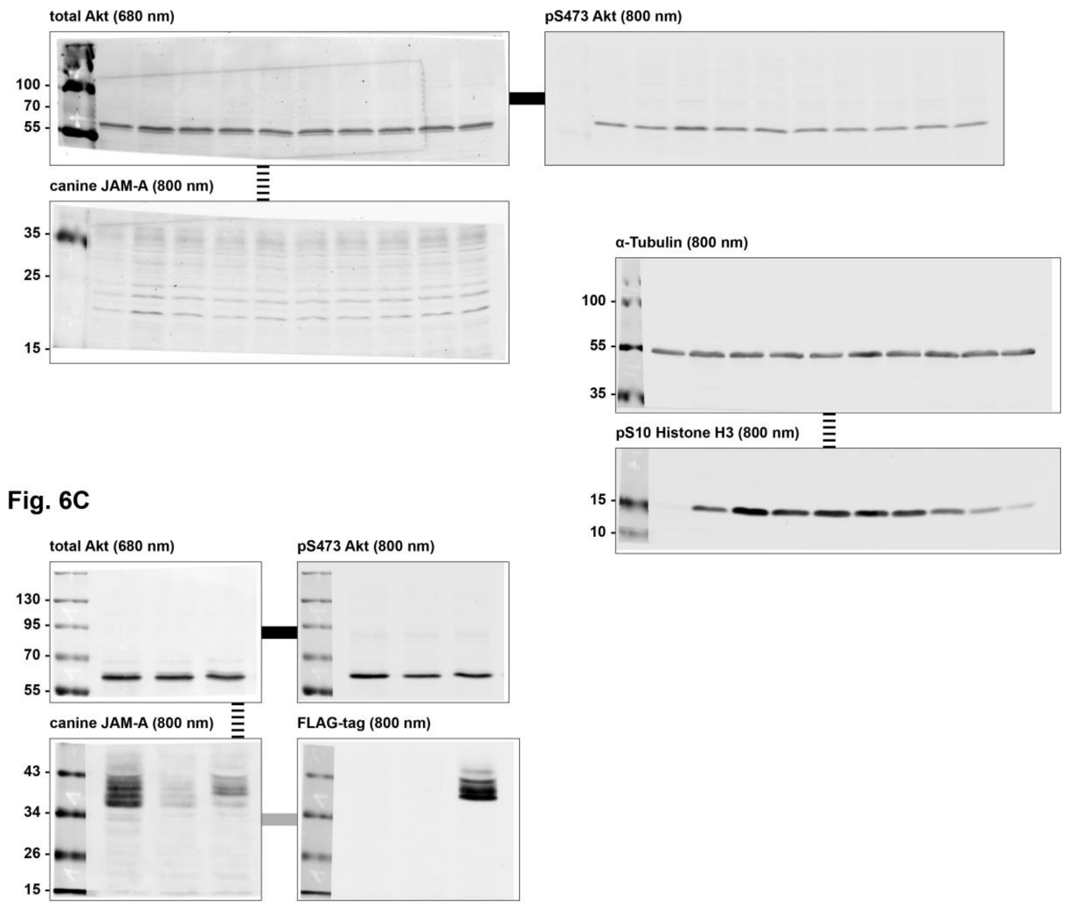

Supplementary Fig. 2A

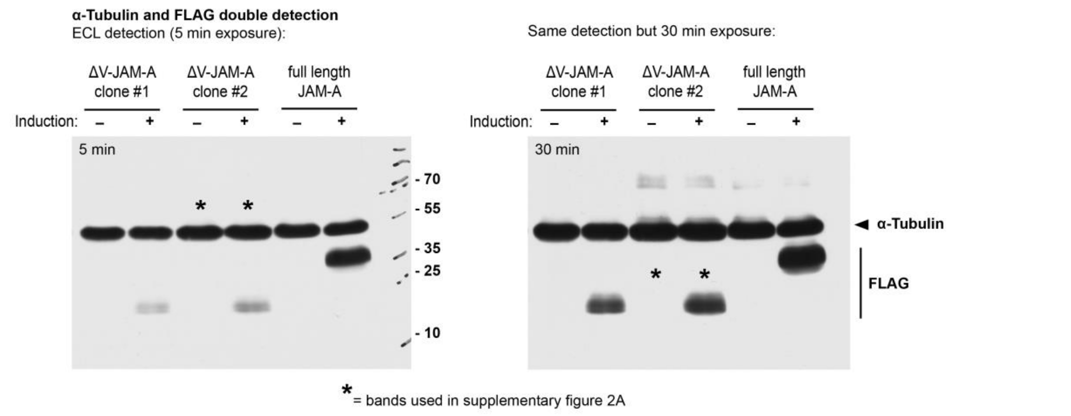

Supplementary Figure 8 continued
